# Depression and its associated factors among textile factory workers at the Almeda textile factory, North Ethiopia

**DOI:** 10.3389/fpubh.2024.1393581

**Published:** 2024-11-21

**Authors:** Tesfaye Derbie Begashaw, Fantahun Andualem

**Affiliations:** ^1^Department of Psychiatry, College of Medicine and Health Science, Aksum University, Axum, Ethiopia; ^2^Department of Psychiatry, College of Medicine and Health Science, University of Gondar, Gondar, Ethiopia

**Keywords:** depressive symptoms, depression, associated factors, textile factory workers, textile factory, Ethiopia

## Abstract

**Background:**

Depression is becoming a significant burden and a potential cause of disability worldwide. According to a World Health Organization (WHO) study conducted in both developed and developing countries, the prevalence of depression is 10.4%. There are differences in the prevalence of depression among the population based on a number of variables, including concomitant medical problems, substance abuse, employment environment, and demographics. The purpose of this study was to evaluate the prevalence of depression and its associated risk factors among textile industrial workers.

**Methods:**

A cross-sectional study was conducted on 409 workers from the Almeda Textile Factory in Adwa from May to June 2020. The outcome variable was depression, assessed using the Patient Health Questionnaire-9 (PHQ-9). Variables with *p*-values of <0.25 in the bivariate analysis were included in the multivariate analysis. An adjusted odds ratio with a 95% confidence interval was computed. Statistical significance was determined at a *p*-value of <0.05.

**Results:**

The total prevalence of depression at a PHQ-9 cutoff score of 10, PHQ-9(10+), was 24.4%. At a cutoff score of 5, PHQ-9(5+) was 51.1%. In the multivariate analysis, low social support, working rotating day and night shifts, and having a physical injury at the workplace were significantly associated with depression at both PHQ-9(5+) and PHQ-9(10+). In addition, advanced age of 45 years and above and being diagnosed with chronic medical disease were significantly associated with depression at PHQ-9(10+) (*p* < 0.05).

**Conclusion:**

The prevalence of depression varied based on the PHQ-9 cutoff score, and it is high at both PHQ-9(5+) and PHQ-9(10+). Low social support, working rotating day and night shifts, physical injury at the workplace, advanced age, and chronic medical disease were significantly associated with depression. Our findings suggest that the workers at the Almeda Textile Factory should be screened and managed accordingly.

## Introduction

Depression, one of the most prevalent mental illnesses, is characterized by a depressed (sad, empty) or irritated mood, a loss of interest or pleasure, and physical and cognitive abnormalities that have a significant impact on a person’s day-to-day functioning (1–4). Depression is becoming a serious burden and a potential cause of disability worldwide (5–7). According to the World Health Organization (WHO), depression will be the second leading cause of disability-adjusted life years (DALYs) by 2030, following HIV/AIDS (8).

A study carried out by the WHO in both developed and developing countries revealed that the prevalence of depression is 10.4% (3). However, the prevalence of depression varies among the global population by area and by high-, middle-, and low-income countries; in the majority of these countries, the prevalence ranges from 3 to 16.9% (9). The prevalence of depression among the population varies depending on a number of variables, including concomitant medical problems (particularly chronic disorders), substance abuse, employment environment, and demographic factors such as gender, age, domicile, marital status, and educational level (1, 3, 4).

Nowadays, life in factories is undoubtedly rather stressful. The range of materials, techniques, equipment, and components used in the textile industry makes it one of the most valuable and technologically advanced sectors overall (10). The pressure and workload on workers in this industry have increased due to the development of new technologies and increased global competition (11). Research has shown that stress at work, a precursor to depression (3), is commonly experienced by workers in textile factories (12, 13). As depression is more prevalent than other mental illnesses, it is well established that it significantly negatively impacts motivation, productivity, absenteeism, and job retention in the workplace (14).

As previously mentioned, there is a risk factor for depression related to gender because women are twice as likely as men to experience depression (1, 3, 4). The majority of workers in textile factories are women (15–20), but little is known about depression in that industry. According to a study, the prevalence of depression varies by age in the United States, with those aged 18 to 29 experiencing a 3-fold higher prevalence than those aged 60 and older (1). According to several research in Ethiopia, the majority of textile factory workers are under the age of 30 (15, 16). According to research, the majority of garment industry workers in Bangladesh have only completed primary school (17) and are single (18).

Acute or chronic physical disease can have an impact on the social, psychological, or biological aspects of health, potentially leading to depression (3). The study was conducted in Myanmar; among textile industry workers, the prevalence of chronic disease was 18.6% for hypertension, 2.2% for diabetes mellitus (19), and 22.3% for hypertension in India (21). Although using personal protective equipment (PPE) might reduce the risks of physical hazards and accidents that textile factory workers face on the job (16, 17), 41.8% of Ethiopian textile industry workers did not wear PPE (22). As a result, those who experience physical injuries may also exhibit depressive symptoms. In fact, given the nature of the condition, depression may even contribute to physical injuries by diminishing concentration and energy. Alcohol and cigarettes have also been reported to be used by textile plant workers (19), and as previously mentioned, substance users tend to have higher levels of depression.

In Ethiopia, the prevalence of depression varies depending on the research population. For example, it was found to be 17.5% in a community-based study (23), 22.9% among university staff (24), 2.2% among students (25), and 48.9% among outpatients (26). As a result, depression varies across populations. Nevertheless, little research has been conducted on the prevalence of depression and the factors associated with it among textile industry workers in Ethiopia and other countries. Consequently, the purpose of this study was to evaluate the prevalence of depression and its risk factors among textile workers in the Almeda area. In addition, the study aimed to address existing gaps in the literature and provide health policymakers, program planners, and strategists with baseline data for the prevention and intervention of depression.

## Methods and materials

### Study area and setting

In May and June 2020, a cross-sectional study with an institutional focus was conducted in the Almeda textile industry in Adwa, Tigray, Ethiopia. The factory is located 1,006 km from Addis Ababa, the capital of Ethiopia, in the center of the Tigray region. The factory was built on 550,000 square meters of land and was founded in February 1996 E.C. Its sub-departments include spinning, weaving, woven processing, knit manufacturing, knit dyeing, and garment production, with fully functional mechanical and electrical workshop equipment. With 4,567 employees, the factory has been sustainably producing goods for more than 25 years, providing employment opportunities in the process.

### Study population

The study population included employees from the textile factory in Almeda who were working in the study area during that time. Workers at the Almeda textile mill with more than 6 months of work experience were included in the study, while those on annual leave, seriously ill, or diagnosed with a mental health diagnosis were excluded.

### Sample size determination

Since no comparable study on depression among Almeda Textile Factory workers has been conducted in our country, the sample size was determined using a single population proportion formula, taking into account the following factors: a standard normal distribution (*z* = 1.96), a 95% confidence interval (*α* = 0.05), a prevalence (*p* = 50%), and a margin of error (d = 0.05). The sample size was 423 when a 10% non-response rate was applied. Using their workplace identification numbers, a computer-generated randomization technique, known as simple random sampling, was used to select study units from among textile industry workers throughout the study.

#### Sampling technique and procedures

**Figure 1:**
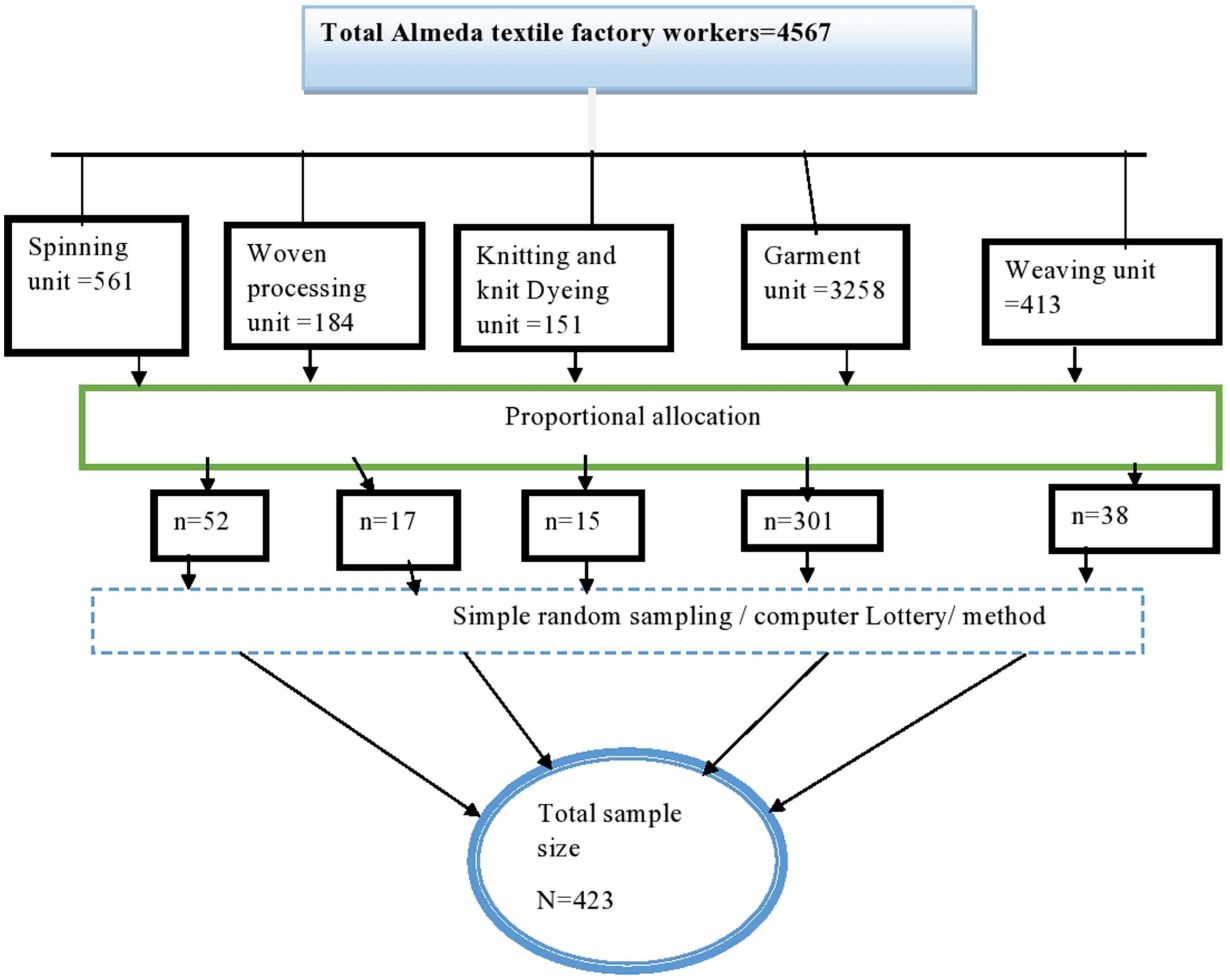
Schematic presentation of proportional allocation to find the required sample among Almeda textile factory workers in Adwa, Tigray, Ethiopia, 2020.

During the study period, the workplaces of textile industry workers were used to select the study unit through simple random sampling (using computer-generated randomization). A sample was chosen from five units of Almeda textile manufacturing workers using proportionate allocation, as illustrated in Figure 1.

### Data collection instruments

The outcome variable, depression, was assessed using the Patient Health Questionnaire-9 (PHQ-9). PHQ-9 cutoff scores of 5 and 10 were used to differentiate the severity grades of depression. The total PHQ-9 score ranges from 0 to 27, with the following cutoff scores: 1–4 indicating “minimal depression,” 5–9 indicating “mild depression,” 10–14 indicating “moderate depression,” 15–19 indicating “moderately severe depression,” and 20–27 indicating “severe depression” (27). In previous studies in Ethiopia, the screening tool was validated at a cutoff point of 10 (27, 28) and at cutoff points of 5 and 10 (29).

Social support was measured using the Oslo Social Support Scale (OSSS-3). The sum score ranges from 3 to 14, with cutoff scores classified as follows: 3–8 indicating poor social support, 9–11 indicating moderate social support, and 12–14 indicating strong social support (30). When categorized into two levels, patients who scored less than 9 were classified as having low social support, and patients who scored 9 or more were classified as having high social support. Work-, clinical-, and substance-related factors were assessed through yes/no answers from the respondents, and socio-demographic background information (age, gender, marital status, occupation, and others) were collected using structured questions.

### Data collection procedure

To ensure consistency and tool understandability, data were gathered through self-administered interviews with the Tigrigna version of a pre-tested questionnaire, which was then translated back into English. Four data collectors (psychiatric professionals with a bachelor’s degree) and two supervisors (master’s degree holders in mental health) received training on data collection instruments, ethical consent, sampling techniques, and protocols. At the apparel velocity corporation (AVC), a pre-test was conducted on 5% of the sample size before the actual data collection. The supervisors oversaw the process, and the completed questionnaires were reviewed for consistency and completeness throughout the data collection process.

### Data processing and analysis

For statistical analysis, the data were imported into Windows’ EpiData Manager version 4.6 and then exported to Statistics Packages for Social Sciences version 22.0. We generated descriptive statistics using the mean, frequency, and percentage. A collection of independent factors and dependent variables was statistically associated using binary logistic regression. In the multivariate analysis, variables from the bivariate analysis with *p*-values less than 0.2 were included. To ensure model fit, the Hosmer–Lemeshow test was performed, and an adjusted odds ratio with a 95% confidence interval was calculated. Statistical significance was declared at a p-value of <0.05.

### Ethical consideration

The Mekelle University College of Medicine and Health Science (MUCHS) office of the health research ethical review committee accepted the study, which was carried out in accordance with the Declaration of Helsinki. MUCHS provided official letters of support, which Almeda Textile Factory (ATF) received and filed. A study participant was made aware of the protocols and importance of the research. Participants’ signed informed consent was acquired. All information, including the results of the analysis, was kept private and shared exclusively with the relevant authorities. Participants were advised that they had the option to withdraw from the study at any time, and those who were not volunteers were not required to take part in it. No personal identifiers were used on the questionnaire, and confidentiality was guaranteed for all the information submitted.

## Results

### Prevalence of depression among the socio-demographic characteristics of the study participants

A total of 409 participants responded to the questionnaire, with a response rate of 96.7%. The mean age of participants was 28.49 ± 7.49 years. More than half of the participants were women (63.6%), while 55.5% were single. Regarding ethnicity, all participants identified as Tigrayan. Among the participants aged 45 years and more, more than 16 (69.6%) participants experienced depression at a cutoff score of 5 on the PHQ-9, PHQ-9(5+), compared to those under 45 years, where 10 participants (43.5%) experienced depression at a cutoff score of 10 on the PHQ-9, PHQ-9(10+). The PHQ-9 diagnosis of depression at a cutoff score of 5 was relatively higher among the women (52.7%) than among the men (48.3%). At a cutoff score of 10, the prevalence was higher among women than men. The prevalence of depression, as measured using the PHQ-9 at cutoff scores of 5 and 10 among divorced or widowed participants, was 57.9 and 47.4%, respectively (Table 1).

**Table 1:** The PHQ-9 diagnosis of depression at cut-off score five and ten in socio-demographic characteristics of the study participants (*N* = 409).

Variables	Categories	Total participants (*N* = 409)	PHQ-9 diagnosis of depression	
			PHQ-9(5+) *n* (%)	PHQ-9(10+) *n* (%)
Age (years)	18–29	259	136 (52.5)	60 (23.2)
	30–44	127	57 (44.9)	30 (23.6)
	> = 45	23	16 (69.6)	10 (43.5)
Sex	Male	149	72 (48.3)	38 (25.5)
	Female	260	137 (52.7)	62 (23.8)
Marital status	Single	227	113 (49.8)	54 (23.8)
	Married	163	85 (52.1)	37 (22.7)
	Divorced/widowed	19	11 (57.9%)	9 (47.4)
Religion	Orthodox	401	206 (51.4)	99 (24.7)
	Muslin	8	3 (37.5)	1 (12.5)
Ethnicity	Tigrayan	409	209 (51.1)	100 (24.4)
Educational status	Elementary	62	32 (51.6)	18 (29.0)
	Secondary	100	49 (49.0)	23 (23.0)
	Diploma	203	112 (55.2)	49 (24.1)
	Degree and above	44	16 (36.4)	10 (22.7)
Salary per month	<1800	185	97 (52.4)	45 (24.3)
	1800–3,000	114	54 (47.4)	24 (21.1)
	>3,000	110	58 (52.7)	31 (28.2)

“PHQ-9(5+),” Patient Health Questionnaire-9 at cut-off score five; “PHQ-9(10+),” Patient Health Questionnaire-9 at cut-off score 10.

### Prevalence of depression in relation to social support, working conditions, and clinical- and substance-related factors among the study participants

Of the total participants, 193(47.2%) had low social support, 93(22.7%) reported a physical injury in the workplace, and 196(47.9%) had consumed alcohol without medical justification in the last 3 months. Among participants with low social support, 58.5% had depression at a cutoff score of 5, while 33.7% had depression at a cutoff score of 10. The PHQ-9 diagnosis of depression at cutoff scores of 5 and 10 among participants who experienced a physical injury at the workplace was 10.5 and 5.3%, respectively. The prevalence of depression at cutoff scores of 5 and 10 was higher among participants with known chronic diseases and those who had used substances (alcohol, cigarettes, or other drugs) in the last 3 months without medical justification (Table 2).

**Table 2:** The PHQ-9 diagnosis of depression at cut off score five and ten in level of social support, working condition, clinical and substance related factors among study participants (*N* = 409).

Variables	Categories	Total participants (*N* = 409)	PHQ-9 diagnosis depression	
			PHQ-9(5+) *n*(%)	PHQ-9(10+) *n* (%)
Social support in three level	Poor	193	113 (58.5)	65 (33.7)
	Moderate	179	77 (43.0)	26 (14.5)
	Strong	37	19 (51.4)	9 (24.3)
Social support in two level	Low/poor	193	113 (58.5%)	65 (33.7)
	High	216	96 (44.4)	35 (16.2)
Working hours/a week	less than or equal to 48	252	130 (51.6)	55 (21.8)
	greater than 48	157	79 (50.3)	45 (28.7)
Work shift	fixed day time	228	98 (43.0)	44 (19.3)
	fixed night time	6	3 (50.0)	1 (16.7)
	rotating day and night	175	108 (61.7)	55 (31.4)
Working department	Garmenting	289	139 (48.1)	61 (21.1)
	Spinning	51	19 (37.3)	7 (13.7)
	knitting and knight dyeing	17	12 (70.6)	8 (47.1)
	Weaving	36	27 (75.0)	17 (47.2)
	woven processing	16	12 (75.0)	7 (43.8)
Physical injury at workplace	Yes	93	64 (68.8)	41 (44.1)
	No	316	145 (45.9)	59 (18.7)
Diagnosed with psychiatry disorders	Yes	4	2 (50.0)	2 (50.0)
	No	405	207 (51.1)	98 (24.2)
Diagnosed with chronic medical diseases	Yes	42	27 (64.3)	17 (40.5)
	No	367	182 (49.6)	83 (22.6)
Current alcohol use	Yes	196	104 (53.1)	45 (23.0)
	No	213	105 (49.3)	55 (25.8)
Current cigarette smoking	Yes	21	13 (61.9)	9 (42.9)
	No	388	196 (50.5)	91 (23.5)
Current another drug use	Yes	14	8 (57.1)	5 (35.7)
	No	395	201 (50.9)	95 (24.1)

Physical injury at workplace (lose of eyes, cut of leg, arm, finger and other related to his/her work in the workplace); Diagnosed with psychiatry disorders (self-reported he/she has known psychiatry disorder); Co-morbid chronic diseases (self-reported) including one or more of (diabetes mellitus, hypertension, HIV/AIDS, and other chronic diseases); Current substance use means use of one or more of (alcohol, cigarette, and other substances) substance in last three-month for non-medication purpose.

### Prevalence of depressive symptoms among study participants

More than half (64.1%) of the participants reported having little interest or pleasure in doing things over the last 2 weeks, indicating that they were at least bothered by this on several days, more than half of the days, or nearly every day. Seventy-one (17.4%) of the participants had suicidal thoughts on at least one of the following frequencies in the last 2 weeks: several days, more than half of the days, or nearly every day. Among the participants, 63.6% reported that their day-to-day activities were slightly to extremely challenging related to the problems (Table 3).

**Table 3:** Prevalence of PHQ-9 depressive symptoms.

Characteristics	Degree of symptoms	Frequency	Percentage (%)
Little interest or pleasure in doing things	0	147	35.9
	1+	262	64.1
Feeling down, depressed, or hopeless	0	221	54.0
	1+	188	46.0
Trouble falling or staying asleep	0	216	52.8
	1+	193	47.2
Feeling tired or having little energy	0	213	52.1
	1+	196	47.9
Poor appetite or overeating	0	271	66.3
	1+	138	33.7
Feeling bad about self	0	265	64.8
	1+	144	35.2
Trouble concentrating	0	280	68.5
	1+	129	31.5
Moving or speaking slowly	0	275	67.2
	1+	134	32.8
Thoughts of being better off dead	0	338	82.6
	1+	71	17.4
Difficulty of day to day activity related to these problems/symptoms	Not difficult at all	149	36.4
	Somewhat difficult	145	35.5
	Very difficult	97	23.7
	Extremely difficult	18	4.4

“0” (not at all); “1+” (one of the following participants’ response; several days, or more than half of the days, or nearly every day).

### The prevalence of depression among the study participants

In this study, the prevalence of depression varied according to the PHQ-9 cutoff score. At a PHQ-9 cutoff score of 10, 24.4% of the participants had potential depression, with a 95% CI of 20–29%. In contrast, at a PHQ-9 cutoff score of 5, the lowest and optimal cutoff point, the prevalence of depression was 51.1%, with a 95% CI of 46–56% (Table 4).

**Table 4:** Prevalence of PHQ-9 diagnosis depression in different cut off score in the study participants.

Characteristics		Frequency	%	95% CI
Severity measured with PHQ-9 score				
<5	Minimal depression	200	48.9	
5–9	Mild depression	109	26.7	
10–14	Moderate depression	64	15.6	
15–19	Moderately-severe depression	29	7.1	
20+	Severe depression	7	1.7	
PHQ-9 (5+)				
<5	Without depression	200	48.9	
5+	With depression	209	51.1	46–56
PHQ-9 (10+)				
<10	Without depression	309	75.6	
10+	With depression	100	24.4	20–29

### Factors associated with depression

After testing all variables for their association with depression at cutoff scores of 5 (PHQ-9(5+)) and 10 (PHQ-9(10+)) in the bivariate analysis, age, social support, work shift, physical injury at the workplace, and diagnosed chronic disease had a *p*-value of less than 0.2. In addition, educational status had a p-value of less than 0.2 at PHQ-9(5+), and current cigarette smoking status also had a p-value of less than 0.2 at PHQ-9(10+). These factors were included in the multivariate analysis as candidates for multivariable logistic regression at a significance level of *p* < 0.05.

In the multivariate analysis, low social support, working rotating day and night shifts, and having a physical injury at the workplace were significantly associated with depression at both PHQ-9(5+) and PHQ-9(10+). In addition, an advanced age of 45 years or above and a diagnosis of chronic disease were significantly associated with depression at the cutoff score of PHQ-9(10+) (*p* < 0.05). The remaining factors were not significantly associated with depression (*p* < 0.05), as shown in Tables 5, 6, respectively.

**Table 5:** Factors associated with depression at cut-off score five PHQ-(5+).

Variables	Depression		COR (95% CI)	AOR(95% CI)	*p*- value
	Yes	No			
Age (year)					
18–29	136(52.5)	123(47.5)		Ref	
30–44	57(44.9)	70(55.1)	0.74(0.48, 1.13)	0.74(0.47, 1.16)	0.186
> = 45	16(69.6)	7(30.4)	2.07(0.82, 5.19)	1.87(0.70, 4.94)	0.210
Educational status					
Elementary	32(51.6)	30(48.4)	1.87(0.85, 4.12)	1.54(0.65, 3.64)	0.329
Secondary	49(49.0)	51(51.0)	1.68(0.81, 3.48)	1.71(0.79, 3.74)	0.177
Diploma	112(55.2)	91(44.8)	2.15(1.10, 4.23)	1.83(0.89, 3.75)	0.099
Degree	16(36.4)	28(63.6)		Ref	
Social support					
low/poor	113(58.5%)	80(41.5)	1.77(1.19, 2.61)	1.66(1.08, 2.55)	0.021
High	96(44.4)	120(55.6)		Ref	
Work shift					
Fixed day time	98(43.0)	130(57.0)		Ref	
Fixed night time	3(50.0)	3(50.0)	1.33(0.26, 6.71)	1.75(0.32, 9.45)	0.517
Rotating day and night	108(61.7)	67(38.3)	2.14(1.43, 3.20)	1.95(1.28, 2.97)	0.002
Physical injury at workplace					
Yes	64(68.8)	29(31.2)	2.60(1.59, 4.25)	2.20(1.31, 3.67)	0.003
No	145(45.9)	171(54.1)		Ref	
Diagnosed with chronic medical disease					
Yes	27(64.3)	15(35.7)	1.83(0.94, 3.55)	1.83(0.90, 3.71)	0.096
No	182(49.6)	185(50.4)		Ref	

COR, crude odds ratio; AOR, adjusted odds ratio; CI, confidence interval; Ref, reference group.

The Hosmer and Lemeshow Test was used to check the model fitness, and its p-value was > 0.05 (*p* = 0.189).

**Table 6:** factors associated with depression at cut-off score 10 PHQ-(10+).

Variables	Depression		COR (95% CI)	AOR (95% CI)	*p*- value
	Yes	No			
Age (year)					
18–29	60(23.2)	199(76.8)		Ref	
30–44	30(23.6)	97(76.4)	1.03(0.62, 1.69)	1.13(0.61, 2.10)	0.703
> = 45	10(43.5)	13(56.5)	2.55(1.07, 6.11)	3.11(1.09, 8.86)	0.034
Marital status					
Single	54(23.8)	173(76.2)	1.063(0.66, 1.71)	1.18(0.65, 2.13)	0.587
Married	37(22.7)	126(77.3)		Ref	
Divorced/widowed	9(47.4)	10(52.6)	3.065(1.16, 8.10)	2.04(0.68, 6.08)	0.201
Social support					
Low/poor	65(33.7)	128(66.3)	2.63(1.64, 4.20)	2.77(1.65, 4.65)	0.000
High	35(16.2)	181(83.8)		Ref	
Work shift					
Fixed day time	44(19.3)	184(80.7)		Ref	
Fixed night time	1(16.7)	5(83.3)	0.84(0.10, 7.34)	1.83(0.20, 17.09)	0.597
Rotating day and night	55(31.4)	120(68.6)	1.92(1.21, 3.03)	1.64(0.99, 2.69)	0.051
Physical injury at working area					
Yes	41(44.1)	52(55.9)	3.43(2.09, 5.65)	2.60(1.52, 4.44)	0.000
No	59(18.7)	257(81.3)		Ref	
Diagnosed with chronic medical disease					
Yes	17(40.5)	25(59.5)	2.33(1.20, 4.52)	2.20(1.05, 4.59)	0.037
No	83(22.6)	284(77.4)		Ref	
Current cigarette smoking					
Yes	9(42.9)	12(57.1)	2.45(1.00,6.00)	2.02(0.70,5.78)	0.191
No	91(23.5)	297(76.5)		Ref	

The Hosmer and Lemeshow Test was used to check the model fitness, and its p-value was > 0.05 (*p* = 0.993).

## Discussion

The overall magnitude of depression varied depending on the PHQ-9 cutoff score. The prevalence of depression at a PHQ-9 cutoff score of 5 was more than 2-fold that of depression at a cutoff score of 10. This finding is consistent with a previous study conducted in Ethiopia (29) and higher than that of an earlier study conducted among healthcare professionals in Ethiopia (31). Furthermore, our search revealed that there were few studies on the prevalence of depression at a PHQ-9 cutoff score of 5 in the same study population. Therefore, we focused our discussion on the PHQ-9 cutoff score of 10. The prevalence of depression at a PHQ-9 cutoff score of 10 was 24.4%, which aligns with studies conducted in Ethiopia (24, 32) and Bangladesh (33). However, this finding was lower than that of previous studies conducted in Ethiopia among university students (25) and in Vietnam (34), while it was higher than that of studies conducted in Ethiopia (35), Saudi Arabia (36), and Vietnam (37). The differences might be related to the study participant, study area and setting, and assessment tool used.

This study revealed the risk factors of depression at PHQ-9 cutoff scores of 5 (PHQ-9(5+)) and 10 (PHQ-9(10+)), which were analyzed during a multivariable logistic regression analysis. In the multivariate analysis, low social support, working rotating day and night shifts, and physical injury at the workplace were significantly associated with depression at both PHQ-9(5+) and PHQ-9(10+). In addition, an advanced age of 45 years and above and a diagnosis of chronic disease were significantly associated with depression at the cutoff score of PHQ-9(10+) (*p* < 0.05). The remaining factors were not significantly associated with depression (*p* < 0.05).

When social support was categorized into two—low and high—low social support was significantly associated with depression, which is consistent with earlier studies conducted in Vietnam (36). However, this finding is not supported by another study conducted in Ethiopia (31). Working in rotating day and night shifts was significantly associated with depression compared to working during the day. Shift work disrupts the circadian clock and affects sleep, which can lead to depression (3). Physical injury at the workplace was significantly associated with depression as compared to the absence of physical injury. Physical injury affected 42.7% of textile factory workers in Ethiopia (16). Loss of an eye, amputation of a leg or arm, and loss of a finger all have psychological and social impacts that may lead to depression (3).

In this study, regarding age, advanced age (45 years and above) was significantly associated with depression at PHQ-9(10+). Advanced age is a risk factor for depression (3), although this finding is not supported by other studies (24, 31, 34, 36). Self-reported known chronic disease was significantly associated with depression at PHQ-9(10+) when compared to no known diagnosis of chronic disease. Chronic disease is directly (biological: HIV/AIDS) or indirectly (psychological and social) a risk factor for depression (3); however, this finding is not supported by a previous study conducted in Ethiopia (A. Belete & Anbesaw, 2022; Yeshaw et al., 2017).

### Limitations

There might have been limitations in establishing a causal relationship between the risk factors and depression due to the cross-sectional study design. In addition, the sample size calculation method used in this study might not support conclusions drawn from an analytical method.

## Conclusion

The prevalence of depression varies according to the PHQ-9 cutoff score. The prevalence of depression at a PHQ-9 cutoff score of 5 was more than 2-fold compared to that of depression at a cutoff score of 10, indicating a high prevalence at both cutoff scores. Low social support, working rotating day and night shifts, physical injury at the workplace, belonging to the advanced age group of 45 years and above, and having a chronic disease were all significantly associated with depression. This study may provide valuable information to concerned stakeholders for the early screening and management of depression among textile factory workers.

## Data Availability

The datasets presented in this study can be found in online repositories. The names of the repository/repositories and accession number(s) can be found in the article/supplementary material.
